# Milk lactose protects against porcine group A rotavirus infection

**DOI:** 10.3389/fmicb.2022.989242

**Published:** 2022-08-17

**Authors:** Xiaolei Ren, Waqar Saleem, Robin Haes, Jiexiong Xie, Sebastiaan Theuns, Hans J. Nauwynck

**Affiliations:** ^1^Laboratory of Virology, Department of Translational Physiology, Infectiology and Public Health, Faculty of Veterinary Medicine, Ghent University, Merelbeke, Belgium; ^2^PathoSense BV, Lier, Belgium

**Keywords:** rotavirus A, VP8*, oligosaccharides, lactose, enterocyte

## Abstract

Rotavirus A (RVA) is an important pathogen causing acute gastroenteritis in animals and humans. Attachment to the host receptor is a crucial step for virus replication. The VP8* domain is the distal terminal region of the RVA spike protein VP4 (expressed by the P gene) and is important for rotavirus binding and infectivity. Recent studies have indicated a role for non-sialylated glycans, including mucin core 2 and histo-blood group antigens (HBGAs), in the infectivity of human and animal group A rotaviruses. In the present study, we determined if porcine rotavirus-derived recombinant VP8* of the endemic strains 14R103 G5P[6], 13R054 G5P[7], 12R010 G4P[13], 12R046 G9P[23], and 12R022 G2P[27] interact with hitherto uncharacterized glycans. We successfully produced five recombinant GST-VP8* proteins of genotype P[6], P[7], P[13], P[23], and P[27]. The hemagglutination assay showed genotypes P[7] and P[23] hemagglutinate porcine and human red blood cells. In an array screen of > 300 glycans, recombinant VP8* of rotavirus genotype P[6], P[7], and P[13] showed specific binding to glycans with a Gal-β-1,4-Glc (β-lactose) motif, which forms the core structure of HBGAs. The specificity of glycan-binding was confirmed through an ELISA-based oligosaccharide binding assay. Further, 13R054 G5P[7] and 12R046 G9P[23] infectivity was significantly reduced by β-lactose in MA104 cells and primary porcine enterocytes. These data suggest that lactose, the main natural sugar in milk, plays an important role in protecting piglets from enteric viral replication and diarrhea.

## Introduction

Rotavirus is an important pathogen causing diarrhea in animals and humans. High morbidity and mortality are mainly observed during the first weeks after birth. The rotavirus particles have 11 double-stranded RNA segments encoding six structural viral proteins (VP1-VP4, VP6, and VP7) and six non-structural proteins (NSP1-NSP6) (Estes and Cohen, 1989). Based on the VP6 sequence, rotavirus has been classified into 10 different species (A to J) (Mihalov-Kovács et al., 2015; Bányai et al., 2017). RVA, B, and C are the most common genotypes that infect humans and animals, including pigs. Among them, the RVA strains have the highest prevalence in a variety of species and is a main cause of diarrhea in the veterinary world. The rotavirus particle’s outer capsid proteins VP7 (glycoprotein) and VP4 (protease-sensitive protein) induce neutralizing antibodies and based on the genes of these proteins (gene G for VP7 and gene P for VP4), a dual classification system has been installed (Matthijnssens et al., 2009). In total, there are 27 different G genotypes and 37 P genotypes reported for RVAs both in animals and humans (Matthijnssens et al., 2011; Trojnar et al., 2013). RVAs with G3, G5, G9 and G11 in combination with P[5], P[6], P[13], P[23], P[27] and P[28] are considered the most common in the world (Matthijnssens et al., 2009; Wang et al., 2010; Okitsu et al., 2011; Theuns et al., 2014). In Europe, America, Africa, and Asia, G5P[6], G5P[7], G9P[13], G9P[23], and G11P[27] have been reported to be the main virus types circulating in piglets (Midgley et al., 2012; Shi et al., 2012; Saikruang et al., 2013; Theuns et al., 2014; Amimo et al., 2015).

The VP4 protein, encoded by the P gene is responsible for viral attachment and is cleaved by a protease into VP8* and VP5*. RVAs of animals and humans have been classified into five P genogroups (I to V) according to the VP8* sequences (Liu et al., 2012). The P[1], P[2], P[3], and P[7] strains are neuraminidase-sensitive strains that belong to genogroup P[I]. The three major human RVA strains P[4], P[6], and P[8], are all classified in P[II]. P[III] contains P[9], P[14], and P[25], which infect both animals and humans. Most avian rotaviruses such as P[17], P[30], P[31], and P[35] belong to P[V] (Liu et al., 2012; Böhm et al., 2015). In former studies, VP8* has been identified to interact with cell surface glycans to initiate viral infection in a genotype-dependent manner (Dormitzer et al., 2002b; Haselhorst et al., 2009; Huang et al., 2012). Many glycans, such as mucin cores, type I HBGA, H1 precursor, human milk (HM) sugars, including lacto-N-tetraose (LNT), lacto-N-fucopentaose I (LNFP 1) were identified as ligands of VP8* to initiate virus attachment (Huang et al., 2012; Liu et al., 2016; Gozalbo-Rovira et al., 2019; Sun et al., 2020). VP8* from human P[6] and P[19] strains bind to H type-1 antigen, which is not the case for porcine P[6] and P[19] strains (Sun et al., 2018). The oligosaccharides from HM sugars were able to inhibit the replication of porcine RVA OSU strain *in vitro* (Hester et al., 2013). These findings indicate that the interaction between P genotypes and cell surface glycans is different among RVA strains and animal species.

In the present study, the recombinant VP8* proteins of five common porcine RVA strains (14R103 G5P[6], 13R054 G5P[7], 12R010 G4P[13], 12R046 G9P[23], and 12R022 G2P[27]) were produced and their binding capacity to cell surface carbohydrates were investigated.

## Materials and methods

### Rotavirus A VP8 genetic analysis

Phylogenetic analysis of 48 Belgian strains was performed based on their VP8* sequences. The phylogenetic tree was constructed using Mega software (v7.0) with the Maximum-likelihood method, reliability of the tree was assessed by bootstrap analysis with 500 replications. Nodes that are supported by bootstrap values higher than 70 are indicated.

### Viruses and cells

Rotavirus strains RVA/Pig-tct/BEL/13R054/2013/G5P[7] (GenBank accession number KF614095.1) and RVA/Pig-tct/BEL/12R046/2012/G9P[23] (GenBank accession number KM820720.1) originating from porcine fecal samples, were passaged 3 times on MA104 cells.

### Expression and purification of VP8* protein in *Escherichia coli*

The VP8* gene segment of the porcine strains RVA/Pig-wt/BEL/14R103/2014/G5P[6] (GenBank accession number KP836271.1), RVA/Pig-wt/BEL/13R054/2013/G5P[7] (GenBank accession number KF614095.1), RVA/Pig-wt/BEL/12R010/2012/G4P[13] (GenBank accession number KF614077.1), RVA/Pig-wt/BEL/12R046/2012/G9P[23] (GenBank accession number KM820720.1) and RVA/Pig-wt/BEL/12R022/2012/G2P[27] (GenBank accession number KF614081.1) originating from porcine fecal samples were extracted and amplified by One-Step RT-PCR with primers (Table 1). The VP8* gene (65–232aa) was cloned into a pGEX-4T-2 expression vector which has a glutathione S-transferase (GST) tag. The correctly identified clones were transformed into *Escherichia coli* (*E. coli*.) strain BL21 (DE3). When the growth of the bacteria reached the logarithmic phase (OD450 ≥ 0.6), the expression of the protein was induced with isopropyl-β-D-thiogalactopyranoside (IPTG) with a final concentration of 0.5 mM at 30°C for 4 h. Glutathione Sepharose 4 Superflow Agarose (Thermo Fisher Scientific) was used to purify the recombinant protein. Briefly, the bacterial suspension was ultrasonicated for 1 min on ice, centrifuged at 4,000 × g for 30 min and the supernatant was collected and run over a glutathione-Sepharose column for 2 h at 4°C. After three times washing with ice cold washing buffer (50 mM Tris-HCl, pH 8.0), the GST fusion protein was eluted with elution buffer (10 mM reduced glutathione, 50 mM Tris-HCl, pH 8.0). Samples of the eluted product were subjected to sodium dodecyl sulfate-polyacrylamide gel electrophoresis (SDS-PAGE) to visualize the expression of the GST-VP8* protein. The GST-VP8* proteins and GST-free protein were desalted by using a 10-kDa concentration tube (Millipore). The level of protein expression was analyzed by SDS-PAGE.

**TABLE 1:** Primer sequences for VP8* amplification.

14R103 G5P[6]-F	GGATCCCCAGGAATTCTAGTACTTGAT
14R103 G5P[6]-R	TCACGATGCGGCCGCAGTATTTATGTA
13R054 G5P[7]-F	CTGGTTCCGCGTGGATCCCTACTGGAT
13R054 G5P[7]-R	CCGGGAATTCTCATAATCCATGATTGA
12R010 G4P[13]-F	CGGAATTCTGTTAGACGGACCATATCAACC
12R010 G4P[13]-R	CACGATGCGCGGCCGCCTAAAGACCATG GTTAAT
12R046 G9P[23]-F	CGCGGATCCCTTGACGGCCCATAT
12R046 G9P[23]-R	CCGGAATTCTCATAATCCATTATTAAT
12R022 G2P[27]-F	CGCGGATCCCTTGATGGACCTTATCAA
12R022 G2P[27]-R	CCGGAATTCTCACAATCCATTATTAAT

### Glycan array screen for glutathione S-transferase-VP8* proteins

The carbohydrate binding specificity of GST-VP8* P[6], P[7], P[13] and control GST-free were determined using a glycan array comprised of 300 glycans (RayBio, GA-Glycan-300). Briefly, the recombinant proteins from different genotypes were used in individual arrays at a concentration of 250 μg/ml and were revealed using a biotinylated anti-GST monoclonal antibody (Thermo Fisher) and Cy3-Streptavidin (RayBiotech). The signals were visualized by a laser scanner (Axon GenePix). To normalize signal intensity data, the sub-array of P[7] was defined as “reference” to which the other arrays were compared by the RayBio Analysis Tool Software. After subtracting background signals and normalization basic on positive controls, signal intensities between and among array images was used to determine relative differences between samples or groups. Any ≥ 1.5-fold increase or ≤ 0.65-fold decrease in signal intensity between samples or groups was considered as a significant difference, provided that both sets of signals were well above the background signal.

### Hemagglutination assay

Porcine red blood cells (RBCs) and human O, A, and B type RBCs (Biobank Red cross-Flanders, M20220623A) were centrifuged for 10 min at 500 x g, and a 0.5% suspension of each RBC type was prepared in 0.85% saline (pH 6.2). 50 μl of the RBCs suspension was mixed with serially diluted GST-VP8* proteins starting from 500 μg/ml in 96-well V-bottom plates (Nunc). The mixture was incubated for 1h at RT and afterward the presence of hemagglutination was analyzed per well.

### Oligosaccharide binding assay

The VP8* proteins were coated in a 96-well microtiter plate at a concentration of 20 μg/well at 4°C overnight. The plates were blocked with blocking buffer (Thermo Fisher, cat: 37516). Synthetic-oligosaccharide-polyacrylamide (PAA)—biotinylated conjugates Lewis^X^ (Gal-β-1,4-(Fuc-α-1,3)GlcNAc, from GlycoNZ), β-lactose (Gal-β-1,4-Glc, from GlycoNZ), cellobiose (Glc-β-1,4-Glc, from GlycoNZ) and L-Rha (from GlycoNZ) were added at a concentration of 0.2 μg/well and incubated at 4°C overnight. Then, 100 μl containing 0.1 μg of HRP-conjugated streptavidin (GE) were added to each well and incubated at 37°C for 1 h. After each step, the plates were washed three times with 0.5% PBS-Tween 20 buffer. Afterward, 100 μl of TMB Substrate Solution (Thermo Fisher, cat: 34021) were added to each well and incubated for 5 min. Finally, 100 μl of 3M H_2_SO_4_ were added to each well and the OD value at 450 nm was determined.

### Blocking rotavirus infection of MA104 cells

Hundred microliters of infectious RVA strains (10^6⋅6^ TCID_50_/ml; treated with 5 μg/ml trypsin) were incubated with either 10 mmol/L or 100 mmol/L β-lactose at 37°C for 1 h in a 24-well plate. The cells were washed with DMEM three times before inoculation. Afterward, the lactose/virus mixtures were transferred on top of MA104 cells, grown in wells of a 24 well-plate with inserts and further incubated at 37°C for 1 h. The inoculum was removed and replaced by MEM containing 100 U/ml Penicillin (Continental Pharma, Puurs, Belgium), 0.1 mg/ml streptomycin (Certa, Braine l’Alleud, Belgium), 0.1 mg/ml gentamycin (Gibco BRL, Merelbeke, Belgium) and 1 μg/ml trypsin. Twelve hours later, the supernatant was collected and inserts with cells were fixed with 4% paraformaldehyde for 10 min and permeabilized with 0.1% Triton X-100 for 5 min at RT. Immunostainings were performed with polyclonal guinea pig anti-monkey rotavirus SA-11 VP6 antibodies (Hoshino et al., 1984) (kindly provided by Prof. John Patton) and goat anti-guinea pig-IgG FITC labeled antibodies (Southern Biotech). Staining of nuclei with Hoechst 33342 (Molecular Probes). Five different fields of cells on each insert were randomly selected under the fluorescence microscope (DMRB fluorescence microscope, Leica Microsystems GmbH, Heidelberg, Germany), and the percentage of infected cells was calculated. The supernatants were collected for virus titration. In brief, 50 μl of tenfold virus dilutions (containing 5 μg/ml trypsin, from 10^–1^ to 10^–8^) were brought on monolayers of MA104 cells in 96-well plates. Five days post inoculation, the cells were examined for cytopathic effect (CPE) by light microscopy and the cell culture infectious dose with 50% endpoint (TCID_50_) was calculated using the formula of Reed and Muench (1938).

### Isolation of porcine enterocytes

Ten-centimeter-long segments of the ileum were collected from 3-days old piglets. To isolate the enterocytes, the mucosal side was turned inside out to make it facing outwards. The segments were washed once with flushing buffer containing 10% FCS, 100 U/ml penicillin, 0.1 mg/ml streptomycin, 0.1 mg/ml gentamycin, 0.01% v/v fungizone (Bristol-Myers Squibb, Braine l’Alleud, Belgium) and two times with flushing medium without FCS (100 U/ml penicillin, 0.1 mg/ml streptomycin, 0.1 mg/ml gentamycin, 0.01% fungizone). The ileum was filled with prewarmed Ca^2+^ and Mg^2+^-enriched PBS, supplemented with 100 U/ml penicillin and 0.1 mg/ml streptomycin and closed with surgical clamps at both sides. The gastrointestinal mucosa was digested in DMEM containing collagenase I (0.4 mg/ml) and dispase II (1.2 mg/ml) for 20 min at 37°C. After releasing the PBS, the lumen was refilled and digested again. Subsequently, the digested mucosa was collected by deeply scraping and incubated in DMEM, containing dispase II (1.2 mg/ml) for 10 min whilst pipetting. The enterocytes were centrifuged and resuspended in a separate solution (20 mg/ml D-sorbitol, 2.5% FCS) and centrifuged at 50 x g for 4 min at 4°C to separate single stromal cells. After 10 times, the pellet was resuspended in DMEM and filtered with 100 μm cell strainers (Falcon). The filtered enterocytes were centrifuged and resuspended in DMEM/F12 supplemented with 10% FCS, 100 U/ml penicillin, 0.1 mg/ml streptomycin, 0.1 mg/ml gentamycin, 0.01% fungizone, 10 ng/ml epidermal growth factor (Sigma), 1% insulin-transferrin-selenium-ethanolamine (Gibco, cat: 51500056) and 1% non-essential amino acids (Gibco, cat: 11140) and seeded in roller tubes. After 1 h incubation at 37°C and 5% CO_2_, the enterocytes were ready for inoculation.

### Blocking rotavirus infection of enterocytes by lactose

To evaluate the inhibitory effect of lactose on the infection of enterocytes with RVAs, the enterocytes from the ileum of 3-days old piglets were used. 20 μl of 13R054 G5P[7] and 54 μl of 12R046 G9P[23] activated virus (treated with 5 μg/ml trypsin), with a titer of 10^7^ TCID_50_/ml and 10^6⋅6^ TCID_50_/ml, respectively, were incubated with either 1 ml of 10 mmol/L or 100 mmol/L β-lactose or mock treated at 37°C for 1 h. After incubation, the mixtures were added to enterocytes for 12 h. For checking the enterocyte viability, ethidium monoazide (EMA) staining was performed every 3 h with 200 μl of enterocytes mixed with 20 μg/ml ethidium monoazide (Invitrogen) for 30 min on ice. Then, the cells were exposed to candescent light for 10 min on ice. Cells were centrifuged, resuspended with PBS and cytospinned onto microscope slides. The enterocytes were fixed with 4% paraformaldehyde for 10 min at RT. Nuclei were stained with Hoechst 33342 (Molecular Probes). The slides were analyzed by fluorescence microscopy. The percentage of viable cell clusters were calculated based on the EMA staining results. To analyze the percentage of infected clusters, 200 μl of infected enterocytes were centrifuged by cytospin. The enterocytes were fixed with methanol (Merck) for 10 min at –20°C. Immunostainings were performed with polyclonal guinea pig anti-monkey rotavirus SA-11 VP6 antibodies and goat anti-guinea pig-IgG FITC labeled antibodies. The percentage of infected cells was counted by fluorescence microscopy.

### Statistical analysis

Data were statistically processed by GraphPad Prism 8.0 (GraphPad software, Inc., San Diego, CA, United States) for analysis of Kruskal–Wallis one-way analysis of variance. The data are represented as means with standard deviation (SD) of three independent experiments. *P* < 0.05 was marked with *; *P* < 0.01 was marked with ^**^; *P* < 0.001 was marked with ^***^.

## Results

### Phylogenetic analysis of VP8* gene sequences

**FIGURE 1:**
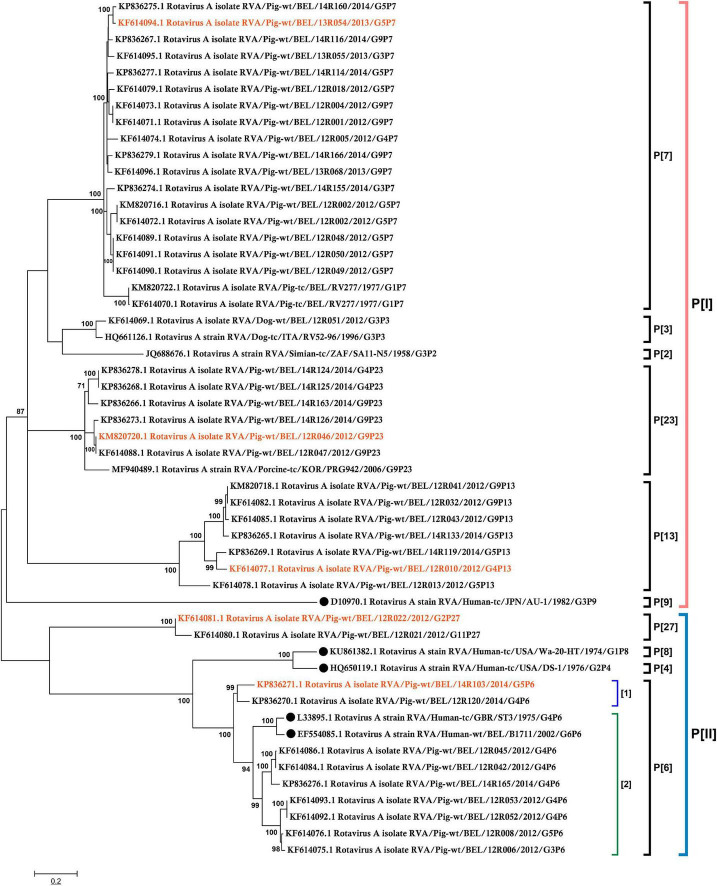
Phylogenetic tree of rotavirus VP8* genes of 48 Belgian porcine RVAs and 5 related human RVAs (indicated by a black dot). The phylogenetic tree was constructed based on the VP8* sequences using the Maximum-likelihood method with bootstrap 500. The bootstrap cutoff value for each clade under 70 is not shown. The red marked RVAs were selected to produce recombinant VP8*.

To select the representative VP8* sequences covering different genetic clusters, a phylogenetic analysis for 48 VP8* sequences from Belgian pig RVAs (Theuns et al., 2015), collected from 2012 till 2014, along with 5 human related RVA reference strains were performed (Figure 1). Based on the phylogenetic tree of the VP8* sequence, 9 genotypes were found. The porcine rotavirus strains were classified into the two genogroups P[I] and P[II]. Genogroup P[I] contained P[3], P[7] (clustered with the human sialic acid-dependent genotypes), P[9], P[13] and P[23] porcine RVAs. The human strain P[9] was genetically far away from the porcine RVAs, with only a genetic similarity of 57%. P[II] included porcine RVAs P[6] and P[27] and the epidemic human genotypes P[4], P[6] and P[8]. The porcine P[6] RVAs have been divided into two subgroups namely [1] and [2]. In subgroup [1], porcine G5P[6] strains were present. Subgroup [2] contains human RVAs P[6] and porcine RVAs P[6]; most of them were combined with G3, G4 and G6. The RVA strains 14R103 G5P[6], 13R054 G5P[7], 12R010 G4P[13], 12R046 G9P[23] and 12R022 G2P[27] were selected to investigate the oligosaccharide binding capacity.

### Production and characterization of recombinant VP8* from 5 porcine rotavirus A strains

**FIGURE 2:**
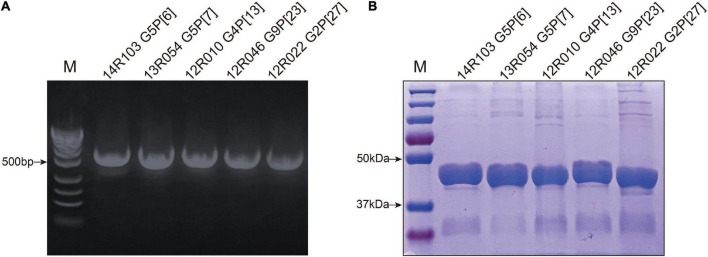
Characterization of recombinant VP8*. **(A)** Amplification of target fragments 498 bp from fecal samples, (lane M = standard molecular weight marker). **(B)** The SDS-PAGE of the GST-VP8* fusion proteins (Lane M = blue prestained protein standard). The 26 kDa band represents free GST.

For the functional assay, the VP8* segments of the selected porcine RVA stains 14R103 G5P[6],

13R054 G5P[7], 12R010 G4P[13], 12R046 G9P[23], and 12R022 G2P[27] were successfully amplified from fecal samples (Figure 2A) and the proteins were expressed as soluble GST fusion proteins (GST-VP8*) in *E. coli*. The molecular weight was approximately 46 kDa (Figure 2B).

### Recombinant GST-VP8* of P[7], P[27], and P[23], but not of P[6] and P[13] hemagglutinate porcine and human blood cells

**FIGURE 3:**
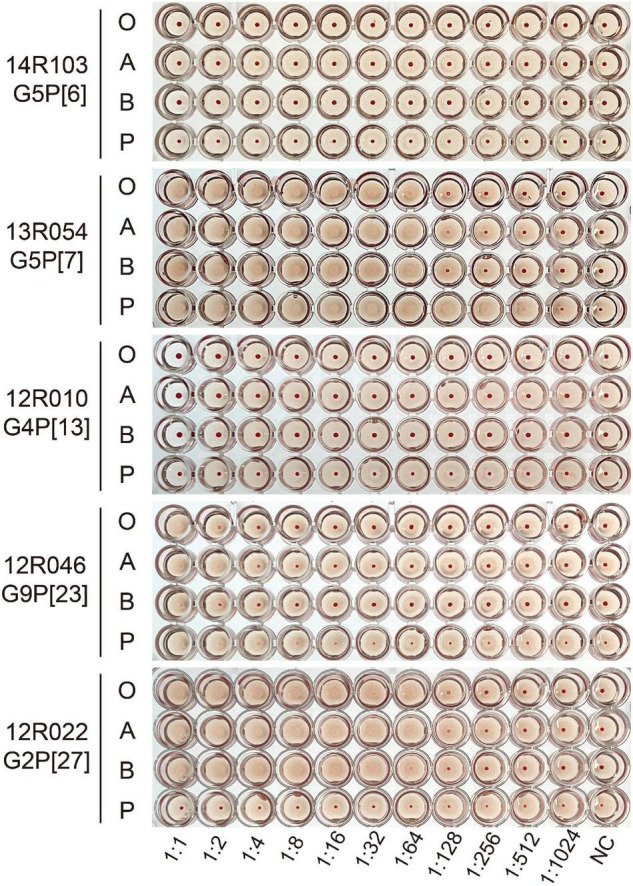
Hemagglutination assays (HA) with the different recombinant VP8* proteins. Human red blood cells of type O, A, and B (marked as O, A, and B), and porcine red blood cells (marked as P) were used. GST-VP8* proteins were serially diluted with PBS and mixed with 0.5% of different types of red blood cells. The PBS buffer without protein was used as negative control (NC).

The recombinant GST-VP8* proteins of five strains were highly expressed in and purified from *E. coli* cultures and analyzed for their hemagglutination capacity. The hemagglutination assay (HA) was performed to test the binding of VP8* protein to human/porcine HBGAs (Figure 3). Hemagglutination occurred with VP8*-13R054 (P[7]) from P[I] (64 hemagglutinating units (HU) with human RBCs and 512 HU with porcine RBCs) and with VP8*-12R046 (P[23]) from P[I] (1–2 HU with human RBCs and 4 HU with porcine RBCs). The hemagglutination of both porcine RBCs and all types of human RBCs indicates that the 13R054 (P[7]) and 12R046 (P[23]) may be sialic acid-dependent strains. VP8* of the 12R022 (P[27]) from P[II] showed hemagglutination with all human RBCs (64 HU), but not with porcine RBCs, confirming that VP8* of P[27] only interacts with certain human specific HBGAs. 12R010 (P[13]) from P[I] and 14R103 (P[6]) from P[II] did not show any hemagglutination. These results indicate that porcine VP8* binds to certain glycans.

### The VP8* of porcine RVA strains 14R103 (G5P[6]), 13R054 (G5P[7]) and 12R010 (G4P[13]) recognize the Gal-β-1,4-Glc (β-lactose) motif

**FIGURE 4:**
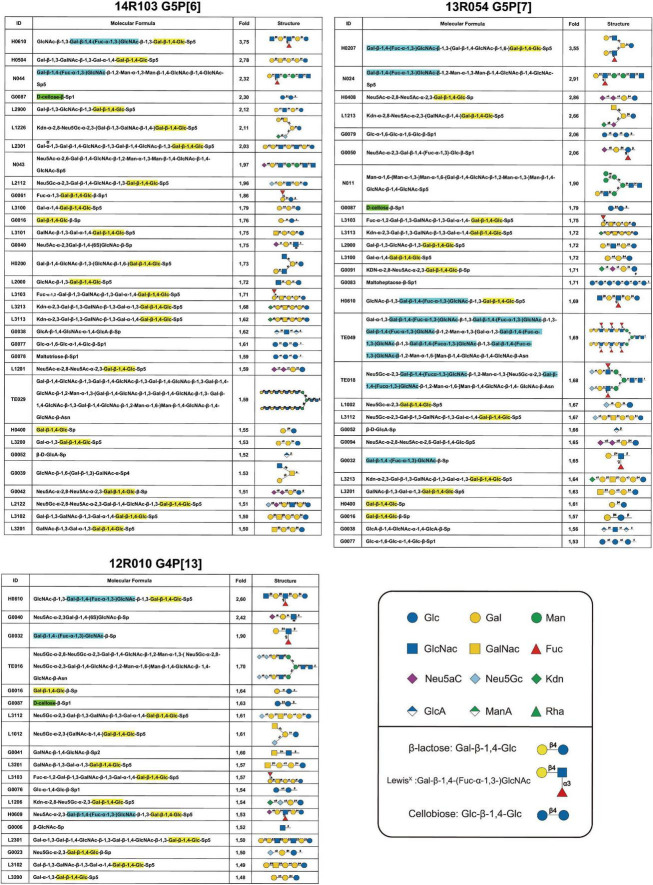
Glycan array results of VP8* proteins from porcine RVA strains 14R103 (G5P[6]), 13R054 (G5P[7]) and 12R010 (G4P[13]). The glycans from an array library containing 300 glycans, that are recognized by each VP8*, were ranked following the binding strength. The values are the fold increase of the fluorescence signal compared to that of GST (negative control). The β-lactose monomer was marked in yellow, the Lewis*^X^* monomer was marked in blue, and the cellobiose monomer was marked in green.

The binding of VP8* from porcine rotavirus RVA strains 14R103 (G5P[6]), 13R054 (G5P[7]) and 12R010 (G4P[13]) to oligosaccharides were carried out by a Glycan-scan (RayBiotech), which tests for 300 sugar groups. After subtracting background signals and normalization to positive controls, relative values (VP8* vs. GST) were obtained and compared. Any ≥ 1.5-fold increase in signal intensity for a single analyte was considered further as a measurable and significant binding [if both sets of signals are well above the background (mean background + 2 standard deviations, accuracy ≈ 95%)]. The oligosaccharides were ranked following the strength of the binding between recombinant VP8* proteins and oligosaccharides (from 3.5- to 1.5-fold-increase). Widely variable glycan binding profiles with top list glycans were detected for individual RVA genotypes (Figure 4). The VP8* proteins reacted mainly with glycans that contain the β-lactose monomer (Gla-β-1,4-Glc, highlighted in yellow), the Lewis*^X^* monomer (Gal-β-1,4-(Fuc-α-1,3-)GlcNAc, highlighted in blue) and cellobiose monomer (Glc-β-1,4-Glc, highlighted in green), which indicates that these three sugars may be involved in the attachment of rotavirus (Figure 4).

### The VP8* recombinant proteins of porcine rotaviruses 14R103 (G5P[6]), 13R054 (G5P[7]), 12R010 (G4P[13]) and 12R046 (G9P[23]) bind to β-lactose

**FIGURE 5:**
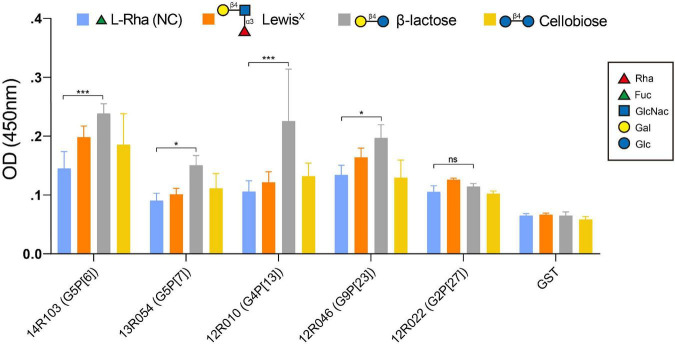
Binding of the recombinant VP8* proteins from the RVA strains 14R103 (G5P[6]), 13R054 (G5P[7]), 12R010 (G4P[13]), 12R046 (G9P[23]) and 12R022 (G2P[27]) to synthetic oligosaccharides. Polyacrylamide (PAA)-biotin conjugates of Lewis*^X^*, β-lactose, cellobiose and L-Rha [negative control [NC)] were used in the binding assay. The GST-VP8* fusion proteins from RVAs 14R103 (G5P[6]), 13R054 (G5P[7]), 12R010 (G4P[13]), 12R046 (G9P[23]), and 12R022 (G2P[27]) were used in the binding assay to confirm the binding specificity. GST-tagged recombinant proteins and the GST tag alone were tested. Error bars in the panel represent the means ± the SD from triplicate experiments. ***, *P* < 0.001. *, *P* < 0.05. ns, *P* > 0.05.

The specificity of the recombinant VP8* binding with β-lactose, Lewis*^X^* and cellobiose was further verified by an oligosaccharide-based binding assay. In comparison with the negative control [L-Rhamnose (L-Rha)], the β-lactose exhibited a significantly higher binding to the recombinant VP8* of the RVA strains 14R103 (G5P[6]), 13R054 (G5P[7]), 12R010 (G4P[13]), and 12R046 (G9P[23]), but not Lewis*^X^* or cellobiose (Figure 5). 12R022 (G9P[27]) showed no binding with any oligosaccharide. These results showed that β-lactose broadly binds with the VP8* of different RVA genotypes.

### β-lactose decreases rotavirus 13R054 G5P[7] and 12R046 G9P[23] infection of MA104 cells

**FIGURE 6:**
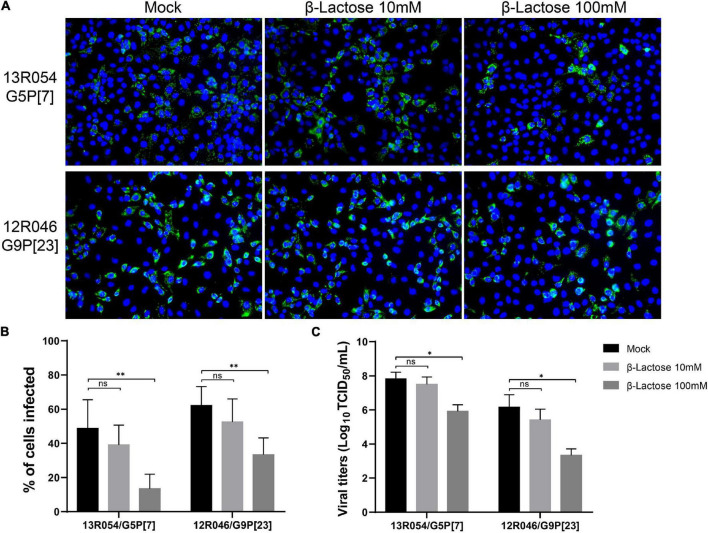
The infection of MA104 cells by the porcine RVA strains 13R054 G5P[7] and 12R046 G9P[23] is strongly reduced by β-lactose. **(A)** The effects of pre-incubation of the virus with β-lactose on the infectivity of the porcine RVAs 13R054 G5P[7] and 12R046 G9P[23] were assessed using an immunofluorescence assay with polyclonal antibodies against the RVA VP6 protein. **(B)** The infection of MA104 cells with both RVA strains was expressed as percentage of infected cells and compared with the mock-treated, virus-inoculated control. **(C)** The titration of the supernatant was performed on MA104 cells. All experiments were performed in three independent experiments. Error bars in the panel represent the means ± the SD from triplicate experiments. ^**^, *P* < 0.01. *, *P* < 0.05. ns, *P* > 0.05.

Based on the VP8* protein- glycan binding assay, we have shown β-lactose can bind with the VP8* of different RVA genotypes. In order to exam if the binding of β-lactose to VP8* protein could block infection of RVAs, the infection blocking assay with the selected β-lactose glycan was performed in MA104 cells. In this assay, only RVA 13R054 G5P[7] and 12R046 G9P[23] were tested as they were the only selected field strains that were able to grow on MA104 cells. Strain passages were minimized to only 3 passages to mimic the *in vivo* wild-type situation as much as possible. A significant dose-dependent decrease in infectivity of both P[7] and P[23] strains was observed by β-lactose (Figure 6). With G5P[7], which is the most prevalent strain, the percentage of infected cells dropped from 48.69 ± 16.83% to 13.45 ± 8.51% and the extracellular virus titer from 10^7⋅8^^±^^0⋅3^ TCID_50_/ml to 10^6⋅0^^±^^0⋅3^ TCID_50_/ml. With G9P[23], the percentage of infected cells decreased from 62 ± 11.09% to 33 ± 9.76% and the extracellular virus titer strongly decreased from 10^6⋅2^^±^^0⋅7^ TCID_50_/ml to 10^3⋅5^^±^^0⋅3^ TCID_50_/ml. These results clearly demonstrate that the β-lactose blocks RVA infection in MA104 cells and suggests that β-lactose may be used as a ligand for infection.

### β-lactose reduces 13R054 G5P[7] and 12R046 G9P[23] infection in primary porcine enterocytes

**FIGURE 7:**
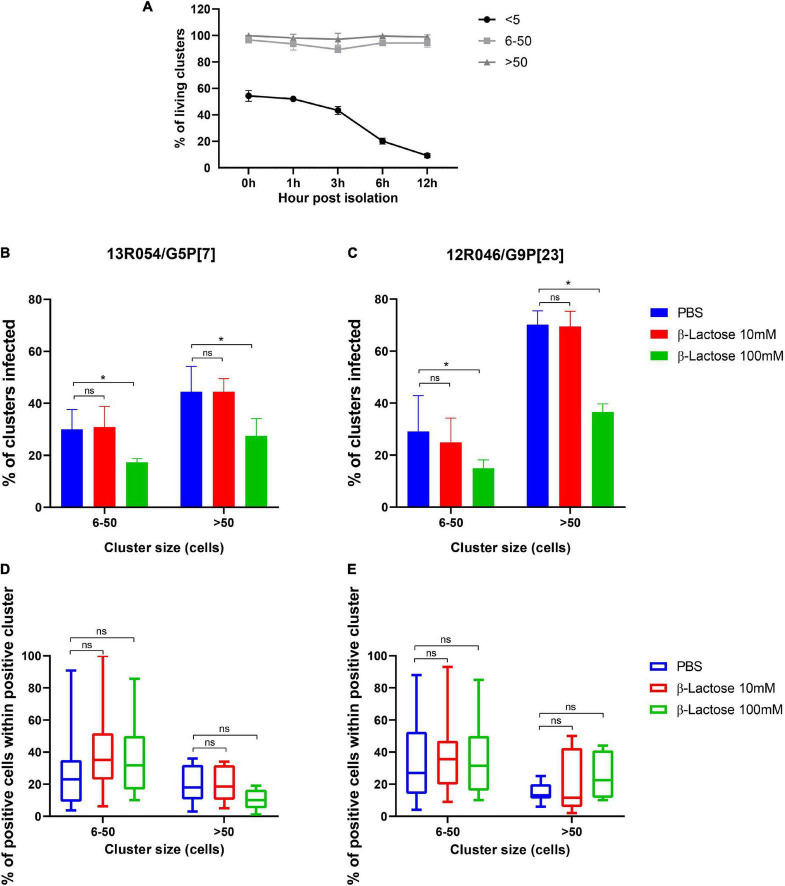
β-lactose pretreatment decreases the replication of rotavirus in porcine enterocytes. After isolation, the ileum enterocytes were incubated with lactose pretreated RVAs for 12 h. **(A)** The viability of clusters during incubation was checked with EMA. The percentage of infected clusters (B-13R054 G5P[7] and C-12R046 G9P[23]) and the percentage of infected cells in a single cluster (D-13R054 G5P[7] and E-12R046 G9P[23]) were determined. **(A–C)** The error bars in the figures represent the means ± the SD from triplicate experiments. **(D,E)** The error bars in the figures represent the min to max. *P < 0.05. ns, *P* > 0.05.

MA104 cells are not the natural target cells for rotavirus. Therefore, the effect of β-glycan was tested in primary enterocytes in parallel. A stable culture of primary porcine enterocytes is necessary to study porcine enteric virus replication characteristics. A new culture method of intestinal epithelial cells has been developed (Cui et al., 2018). Enterocytes were isolated from the ileum of 3-days piglets and directly inoculated with RVA strains 13R054 G5P[7] and 12R046 G9P[23] that had been pre-incubated with 10 mM or 100 mM β-lactose or mock-treated. Clusters were classified and counted according to their size from 0 to 5, 5 to 50, and more than 50 cells in a single cluster. Twelve hours after inoculation, the middle-sized clusters (5–50 cells) and large-sized clusters (more than 50 cells) stayed at a high viability level till 12 hpi, while the viability of single cells and cells in small-sized clusters (less than 5 cells) was decreasing in function of time (Figure 7A). The proportion of clusters with infected cells and the proportion of infected cells within the cluster were calculated. Both RVA 13R054 G5P[7] and 12R046 G9P[23] infected large-sized clusters more efficiently than middle-sized clusters. RVA strain 13R054 G5P[7] showed 31 ± 7.59% infectivity in middle-sized clusters and 44 ± 9.79% in clusters containing more than 50 cells (Figure 7B). The 12R046 G9P[23] infected 29 ± 13.79% of middle-sized clusters and 70 ± 5.24% of large-sized cluster (Figure 7C). β-lactose could notably decrease the infection rate of 13R054 G5P[7] and 12R046 G9P[23] in the middle-sized clusters and large-sized clusters at the 100 mM concentration, but not at the 10 mM concentration (Figures 7B,C). β-lactose did not significantly affect the proportion of positive cells within the positive cluster (Figures 7D,E). These results demonstrated that the β-lactose also reduces rotavirus infectivity in primary porcine enterocytes.

## Discussion

Group A rotaviruses (RVAs) are a major cause of acute diarrhea in animals and humans. The cell-attachment protein VP8* of rotavirus is a target for potential antivirals and receives a lot of attention. Attachment of RVA to enterocytes occurs in the presence of specific glycans including mucin glycans and cell surface glycans (HBGAs and SA) (Yolken et al., 1987; Jolly et al., 2000). Terminal N-acetylneuraminic acid (sialic acid [SA]) is recognized by VP8* proteins of some rotavirus strains for virus attachment as evidenced by a decreased rotavirus infection in neuraminidase pre-treated cells (Ciarlet et al., 2002). Ciarlet et al. tested the SA dependence of 20 P genotypes (P[1]–P[20]) of animal and human rotaviruses. Only animal rotavirus genotypes P[1], P[2], P[3], and P[7] were identified as SA-dependent strains (Fukudome et al., 1989; Ludert et al., 1998; Ciarlet and Estes, 1999; Delorme and Bru, 2001; Ciarlet et al., 2002). This can explain why in our study VP8* of P[7] agglutinated both human and porcine RBCs and VP8* of P[6] and P[13] strains did not. Since VP8* of P[23] also agglutinated all human and porcine RBCs, it most probably also recognizes SA for attachment. This is further supported by a decreased infection of neuraminidase treated MA104 cells (Cui et al., 2019). VP8* of porcine rotavirus P[27] behaved somewhat differently. It agglutinated human RBCs but not porcine RBCs. This indicates that genotype P[27] is evolutionary close to human genotypes in P[II].

Histo-Blood Group Antigens (HBGAs) are the complex oligosaccharides present on the surface of RBCs and mucosal epithelia (Clausen and Hakomori, 1989; Hakomori, 1999). Recent studies have demonstrated the importance of HBGAs as cellular attachment and susceptibility factors for human RV in a genotype-dependent manner (Sun et al., 2020; Guo et al., 2021). Genotypes P[6] and P[19] bind with H1 for human strains but not for porcine strains (Li et al., 2018; Sun et al., 2018). This was also observed in our glycan-scan analysis that demonstrated that VP8* of porcine 14R103 P[6] did not bind with H1. In addition, our data showed that porcine VP8* of 14R103 P[6] could recognize mucin core 2 (GlcNAc-β-1,6-(Gal-β-1,3-)GalNAc), which was also reported with the porcine P[6] strain z84 (Sun et al., 2018). Other oligosaccharides were also identified as targets for porcine rotaviruses. The SA-dependent porcine rotavirus strain OSU P[7] was demonstrated to recognize N-acetylneuraminic acid (Neu5Ac), N-glycolylneuraminic acid (Neu5Gc), and sia-containing compounds (Rolsma et al., 1998; Dormitzer et al., 2002a). Porcine rotavirus strain CRW-8 P[7] showed a binding ability with 3′-SL (Neu5Ac-α-2,3-Gal-β-1,4-Glc) by crystallization (Yu et al., 2011). The results are consistent with our glycan-array results, which demonstrate that VP8* of the porcine rotavirus strain 13R054 P[7] also recognized 3′-SL. Taking these findings together, it can be confirmed that HBGAs, mucins and sialic acids play important roles in porcine infection.

In our glycan-array analysis, Gal-β-1,4-Glc always showed up within the structure of the sugars that interacted with VP8*. In the oligosaccharide binding assay, the recombinant VP8* of the P[6], P[7], P[13], and P[23] strains showed binding with β-lactose. It is remarkable that β-lactose may act as a central ligand for all RVAs. Earlier, 3′-SL (Neu5Ac-α-2,3-Gal-β-1,4-Glc) and 6′-SL (Neu5Ac-α-2,6-Gal-β-1,4-Glc) were reported to inhibit porcine strain OSU (P[7]) infectivity in MA104 cells in a dose-dependent manner with IC_50_ of 6 mM (Hester et al., 2013). In the present study, we found that 100 mM β-lactose (Gal-β-1,4-Glc) can inhibit P[7] and P[23] rotavirus replication on MA104 cells, which suggested a lower concentration of β-lactose was needed when conjugated with Neu5Ac. Interestingly, the pre-incubation of both strains with β-lactose inhibited their infectivity in MA104 cells, suggesting that β-lactose binding may interfere with the SA binding site. Crystallographic studies will be necessary to determine whether the SA and β-lactose binding sites are located on the VP8* domains of both strains.

One must be careful with extrapolating the results obtained in the MA104 continuous cell line. Intestinal enterocytes are the real target for rotavirus infection. We developed a new culture method of intestinal epithelial cells to determine rotavirus infection and examine the blocking effect of oligosaccharides. Only clusters containing more than 5 cells stayed alive for 12 h after isolation. We demonstrated that both porcine G5P[7] and G9P[23] were able to replicate in enterocytes efficiently. Interestingly, the number of infected clusters decreased with the increasing concentration of lactose, indicating that β-lactose could bind with VP8* of viruses and block infection on the outer side of epithelial cells of the clusters. Within the infected clusters, the number of infected cells did not change significantly with the increasing concentration of lactose. This is possibly due to the close cell-cell adhesions and ease of infectious viral particles to directly spread from cell to cell without coming in the extracellular medium. Our new *in vitro* enterocyte model can be used for rotavirus receptor investigation. It elegantly mimics RV infection *in vivo*.

Lactose, a milk-specific reducing disaccharide, is one of the major components of milk in most animal species. Milk typically contains 140 mmol/L lactose, a level that decreases as lactation progresses. The concentration of lactose in the milk of the major dairy species—cows, buffaloes, goats, and sheep—is approximately the same at 130–150 mmol/L (Schaafsma, 2008). Genetical background or diet of the animal has little effect on the lactose content of the milk due to a fixed relationship between lactose concentration and blood osmolarity. In our study, the high concentration of 100 mmol/L of lactose reduced viral infection not only in the generally used MA104 cells but also in the real target cell, the enterocytes. As the concentration of lactose used in the present study was similar to the concentration in milk, one may extrapolate that it might also have an effect *in vivo*. Piglets are usually weaned at 3–4 weeks after birth and RVA-associated diarrhea in piglets is most frequently observed in pigs aged 3–5 weeks (Homwong et al., 2016; Zimmerman et al., 2019). The reason why RVA is circulating and affecting piglets at that age may be explained, on the one hand, by the protective effect of maternal antibodies in milk (lactogenic immunity) as long as the animal is suckling and on the other hand, by lactose that is extremely high during the first weeks of life. In a follow-up study also supports the hypothesis that feeding with lactose improved performance during the 1st-week post-weaning and reduced diarrhea incidence (Pierce et al., 2005).

Enteric co-infections are common and many intestinal bacteria and viruses are involved (O’Ryan et al., 2005). Enteric co-infections may cause more severe diarrhea than single infections (Tzipori et al., 1981; Grimprel et al., 2008). Earlier studies showed that rotavirus-*E.coli* co-infections increased animal morbidity and mortality (Tzipori et al., 1980; Newsome and Coney, 1985). Rotavirus damages the epithelium of the small intestines, which may favor the luminal environment for the colonization of enteropathogenic *E. coli* (Lecce et al., 1982). In the present study, we found that β-lactose could block rotavirus infection. Several studies also reported that lactose may positively modulate the gut microbial community as it specifically changes the metabolites in the environment (Venema, 2012). Dietary lactose has been shown to have a probiotic effect on the microbiota in the gastrointestinal tract of weaned piglets (Charalampopoulos et al., 2002). Microorganisms in the gastrointestinal tract can ferment lactose to lactic acid and volatile fatty acids, which lowers the pH of the gastric environment. A lower pH environment is not conducive to pathogen storage and inhibits the growth of *E. coli* (Bach Knudsen, 2012; Zhao et al., 2019). Therefore, it may be speculated that lactose can improve diarrhea problems in young pigs by a combination of a direct effect by reducing rotavirus infection and inhibiting the growth of bacterial pathogens by balancing the environment.

## Conclusion

In conclusion, it may be stated that lactose is important for young pigs and should be present at high levels in the feed. As it is known that avidity is important for sugar-protein interactions, different complex sugar structures containing multiple lactose oligosaccharides will be tested in the future for their rotavirus-inhibitory activity.

## Data availability statement

The original contributions presented in the study are included in the article/supplementary material, further inquiries can be directed to the corresponding author/s.

## Ethics statement

The animal study was reviewed and approved by the Local Ethical Committee of the Faculty of Veterinary Medicine, Ghent University.

## Author contributions

HN and XR designed the experiments. XR, WS, and RH performed the experiments. XR analyzed data and wrote the manuscript. JX and ST contributed by providing materials. All authors contributed to the article and approved the submitted version.
